# Rheological characterization of poly-dimethyl siloxane formulations with tunable viscoelastic properties†

**DOI:** 10.1039/d1ra03548g

**Published:** 2021-11-05

**Authors:** Thomas J. Petet, Halston E. Deal, Hanhsen S. Zhao, Amanda Y. He, Christina Tang, Christopher A. Lemmon

**Affiliations:** Department of Biomedical Engineering, Virginia Commonwealth University Richmond VA USA clemmon@vcu.edu +1-804-827-0446; Joint Department of Biomedical Engineering, North Carolina State University, University of North Carolina Chapel Hill Raleigh NC USA; Department of Biology, Duke University Durham NC USA; Department of Chemical and Life Science Engineering, Virginia Commonwealth University Richmond VA USA; Comparative Medicine Institute, North Carolina State University Raleigh NC USA

## Abstract

Studies from the past two decades have demonstrated convincingly that cells are able to sense the mechanical properties of their surroundings. Cells make major decisions in response to this mechanosensation, including decisions regarding cell migration, proliferation, survival, and differentiation. The vast majority of these studies have focused on the cellular mechanoresponse to changing substrate stiffness (or elastic modulus) and have been conducted on purely elastic substrates. In contrast, most soft tissues in the human body exhibit viscoelastic behavior; that is, they generate responsive force proportional to both the magnitude and rate of strain. While several recent studies have demonstrated that viscous effects of an underlying substrate affect cellular mechanoresponse, there is not a straightforward experimental method to probe this, particularly for investigators with little background in biomaterial fabrication. In the current work, we demonstrate that polymers comprised of differing polydimethylsiloxane (PDMS) formulations can be generated that allow for control over both the strain-dependent storage modulus and the strain rate-dependent loss modulus. These substrates requires no background in biomaterial fabrication to fabricate, are shelf-stable, and exhibit repeatable mechanical properties. Here we demonstrate that these substrates are biocompatible and exhibit similar protein adsorption characteristics regardless of mechanical properties. Finally, we develop a set of empirical equations that predicts the storage and loss modulus for a given blend of PDMS formulations, allowing users to tailor substrate mechanical properties to their specific needs.

## Abstract 2

We have generated novel formulations of polydimethyl siloxane with varying viscoelastic properties that can be used to study cellular response. We present equations that can be used to predict the storage and loss moduli of these polymers.

## Graphical abstract

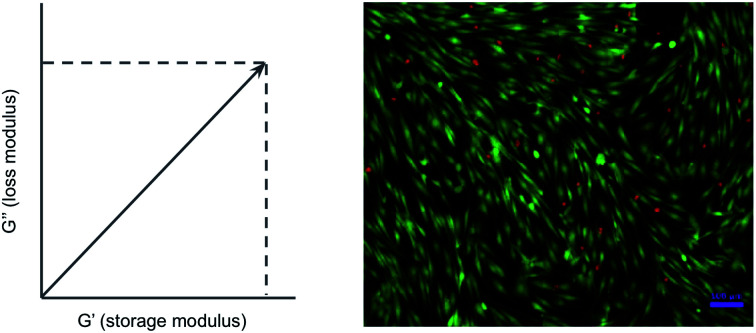

## Introduction

The field of mechanobiology has become a burgeoning field of research. Discoveries that showed that the stiffness (elastic modulus) of substrates can drive cell migration, cell proliferation, and cell differentiation^1–6^ were groundbreaking and created a paradigm where we could envision that the elastic modulus of *in vivo* tissue could be a key player in disease progression. Indeed, studies have shown that this is the case in many pathologies: tumor stiffness correlates with aggressiveness in cancer,^7–10^ liver stiffness correlates with liver dysfunction in patients with chronic liver disease,^11–13^ and increased stiffness in kidneys predict chronic kidney disease earlier than other markers.^14–16^ These findings suggest a significant clinical implication of cellular response to stiffness.

However, there is a significant concern with drawing correlative conclusions from these studies: soft tissue in the human body is viscoelastic,^17–19^ and the majority of mechanobiology research has focused only on the elastic component. Viscoelastic tissues have viscous properties, meaning that the tissue will continue to deform over time for a given load (for example, silly putty, which will continue to stretch over time when exposed to a constant shear force), and elastic properties, where the tissue strain is only proportional to the force applied (*i.e.*, a stretched spring under a constant force, which will stay at a constant displacement, regardless of time). Viscoelastic materials can be quantified by the loss modulus, which represents the viscous component, and the storage modulus, which represents the elastic component. Most tissues in the human body exhibit viscoelastic behavior, with a loss modulus roughly 10 percent of the tissue's storage modulus.^20^ Most soft tissues in the body demonstrate viscoelastic properties that are subcategorized as “strain-stiffening materials”, where as the material stretches, it becomes effectively stiffer.^17–19,21^ Loss moduli have been quantified for various tissues, including lung (∼600 Pa),^22^ brain (∼1 kPa),^23^ cornea (∼12 kPa),^24^ and liver (∼17 kPa),^25^ suggesting that the loss modulus of soft tissues varies greatly throughout the body.

While the majority of work in the field of mechanobiology has only probed the effects of the elastic modulus on cellular response, studies have shown that increases in tissue elastic modulus occurs late in disease progression.^26^ At early stages of fibrotic diseases, where there is significant assembly of *de novo* extracellular matrix, the tissue does not exhibit an increased stiffness, or storage modulus, but does exhibit a significant change in loss modulus.^26^ There has been a growing interest in the role of the loss modulus in modulating cellular response: several studies have probed the effects of altered creep or viscoelasticity in hydrogels and demonstrated that an altered viscous component can affect cell size, focal adhesion formation, and proliferation.^27–30^

The majority of work investigating cellular responses to substrate mechanics have utilized polyacrylamide gels, where the elastic modulus can be regulated by changing the concentration of the bis-acrylamide crosslinker.^31^ Polyacrylamide gels have a loss modulus near zero,^32^ and as such, these studies have examined the cellular response to an almost purely elastic substrate. These gels can results in substrates with elastic moduli ranging from the order of 0.5 Pa to 300 kPa,^33,34^ which does not span the full range of biologically relevant stiffnesses.^35^ Cellular mechanoresponse studies have also utilized PDMS, which is capable of generating surfaces with a much larger range of elastic moduli, ranging from 1 kPa to 1.5 MPa.^36^

While most cellular mechanoresponse work has focused on purely elastic surfaces, a few experimental methodologies have been developed for altering the loss moduli of cell substrates; these have primarily focused on modifying polyacrylamide gels to increase the loss modulus. One approach is to add long linear polyacrylamide chains, which are trapped within the polyacrylamide gel. Substrates generated using this approach exhibit storage moduli in the range of 1 kPa to 6 kPa and loss moduli in the range of 1 to 500 Pa.^27,37^ The loss modulus of polyacrylamide gels can also be altered by fine-tuning of the ratio of acrylamide to bis-acrylamide;^29^ however, this approach results in small changes in the loss modulus (1 to 130 Pa) and can only be utilized for low storage modulus (4 kPa) gels. As such, there is not a currently available approach that can span the ranges of both physiologically relevant storage moduli and loss moduli.

While experimental methodologies exist to vary either the storage modulus or the loss modulus of cellular substrates, there is not an established system to vary both. Here we present a system that allows for control of both, with little to no experience with generating polymer substrates. We have generated surfaces comprised of two formulations of PDMS, namely Sylgard 184 and Sylgard 527, and demonstrate that by changing the base : crosslinker ratio of Sylgard 184 and the ratio of Sylgard 184 to Sylgard 527, we can generate surfaces with a wide range of both storage and loss moduli. These surfaces adsorb protein at similar rates, have comparable biocompatibility, and are easily fabricated in research labs without specific expertise in biomaterials. We have developed empirical equations for the storage and loss moduli as a function of Sylgard 184 base : crosslinker ratio and Sylgard 184 : Sylgard 527 ratio, which will allow researchers to formulate substrates to meet specific experimental needs.

## Results

### Formulation of PDMS blends

PDMS polymers are generated by mixing a base and crosslinker at various ratios and curing at 110 °C. This can be done using either Sylgard 184 or Sylgard 527; both share the same basic chemical structure, with the major difference between the two polymers being the chain length of each polymer: Sylgard 184 has 184 repeated mer units while Sylgard 527 has 527 repeated mer units. While the exact chemical structures of the curing agents in both Sylgard 184 and Sylgard 527 are not known due to the proprietary nature of the polymer, we can hypothesize that polymer chains of PDMS having either 184 or 527 mer units as the base interact with dimethylvinylsiloxy-terminated chains of the same length. Multiple catalyst and filler chemicals interact with the Si=CH_2_ bond randomly along the polymer and form a network of varying chain lengths of PDMS. Previous studies have shown that the elastic modulus of PDMS can be altered by changing either the base : crosslinker ratio of Sylgard 184 PDMS^38^ or by changing the ratio of Sylgard 184 : Sylgard 527.^36^ To the best of our knowledge, no studies have reported the effects of altering the base : crosslinker ratio nor the Sylgard 184 : Sylgard 527 ratio on the loss modulus of substrates, nor have studies investigated the combinatorial effect of altering both the base : crosslinker and 184 : 527 ratios. To investigate the combinatorial effects of these two on surface mechanical properties, we fabricated an array of PDMS blends. The base : crosslinker ratio of Sylgard 184 was varied from 5 : 1 to 30 : 1 (w : w), and the ratio of 184 : 527 was varied from 1 : 5 to 1 : 30 (w : w). Each formulation uses the notation (*X* : 1) : *Y* in which *X* : 1 is the base : crosslinker ratio of Sylgard 184, and *Y* represents the ratio of Sylgard 527 to the Sylgard 184. Sylgard 184 base and crosslinker, and Sylgard 527 base and crosslinker were mixed separately and then degassed under vacuum. The two mixtures were then combined at the appropriate ratio, degassed again, and then cured at 110 °C for 18 h. Note that for all formulations, Sylgard 527 was mixed at a 1 : 1 (w : w) ratio of base : crosslinker as recommended by the manufacturer.

### Quantification of storage and loss moduli

**Fig. 1:**
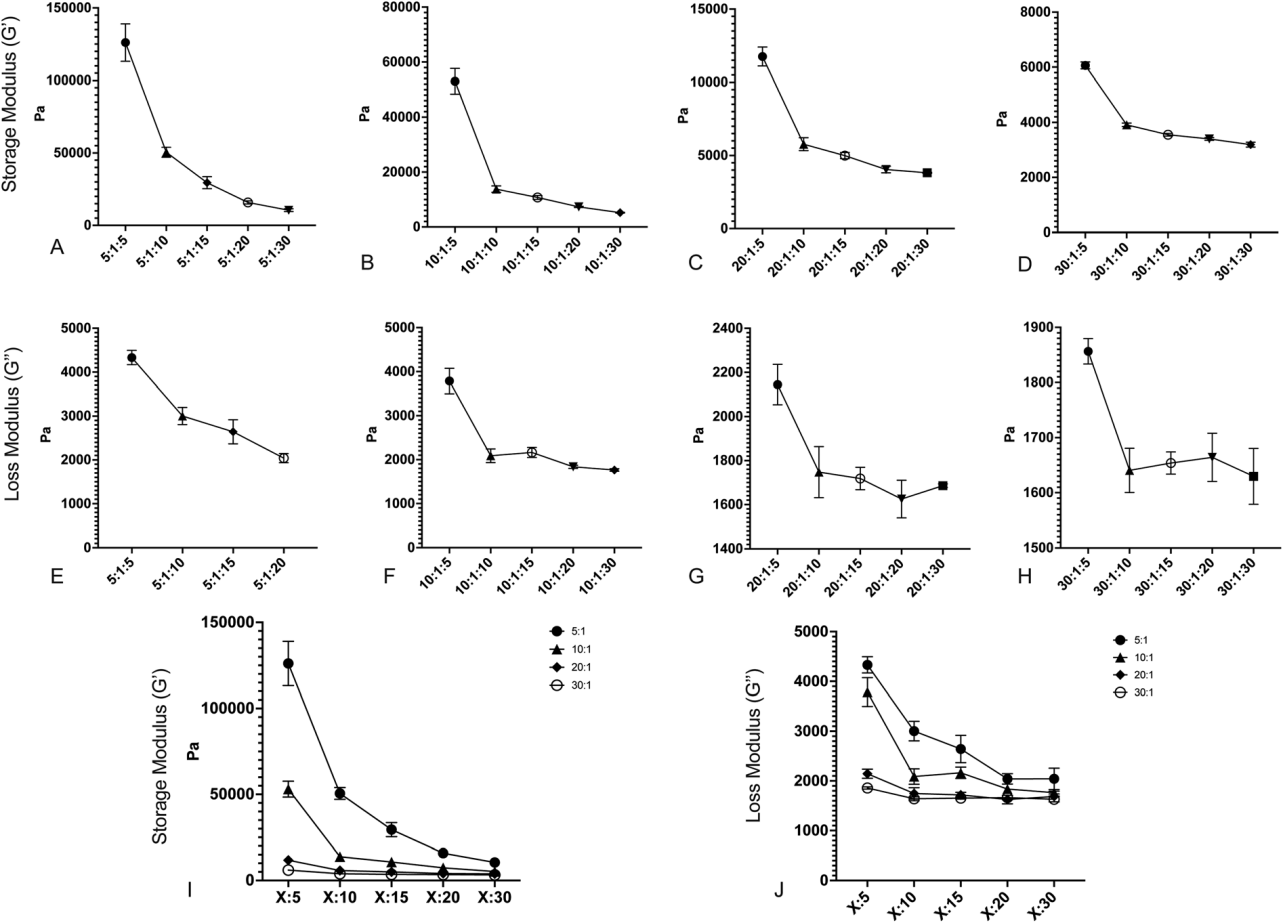
Changing the ratios of Sylgard 184 base : crosslinker and Sylgard 184 : 527 creates surfaces with varying storage and loss moduli. (A–D) Storage modulus (*G*′) *versus* decreasing Sylgard 184 : Sylgard 527 ratio for (A) 5 : 1 B : C Sylgard 184, (B) 10 : 1 B : C Sylgard 184, (C) 20 : 1 B : C Sylgard 184, and (D) 30 : 1 B : C Sylgard 184. (E–H) Loss modulus (*G*′′) *versus* decreasing Sylgard 184 : Sylgard 527 ratio for (E) 5 : 1 B : C Sylgard 184, (F) 10 : 1 B : C Sylgard 184, (G) 20 : 1 B : C Sylgard 184, and (H) 30 : 1 B : C Sylgard 184. (I) Compiled values for storage modulus for all formulations. (J) Compiled values for loss modulus for all formulations. Error bars represent standard error. Formulations are labeled on the *X*-axis according to the notation (184B : 184C) : 527.

After fabricating the substrates, we quantified the storage and loss moduli *via* parallel plate rheometry. Samples with a 20 mm diameter and a height of 5 mm were fabricated from each formulation, with a minimum of 3 samples per formulation. Samples were compressed with a 3 N axial force, and were subjected to 5% oscillating rotational strain over a range of oscillating frequencies from 1 to 100 rad s^−1^. The resulting amplitude and phase shift of the force was measured and converted to values for *G*′ and *G*′′ using the TA Instruments Trios software package. Fig. 1 shows values for *G*′ and *G*′′ for formulations with: a 5 : 1 base : crosslinker 184 ratio (Fig. 1A and E); a 10 : 1 base : crosslinker 184 ratio (Fig. 1B and F); a 20 : 1 base : crosslinker 184 ratio (Fig. 1C and G); and a 30 : 1 base : crosslinker 184 ratio (Fig. 1D and H). All values shown are at 100 rad s^−1^ oscillation frequency; while cells develop traction forces slowly, cellular filopodia rapidly apply forces to underlying substrates, and as such, we have focused on the high frequency response. Full data for all frequencies are shown in Fig. S1.† Results indicate that both storage modulus and loss modulus of the formulations decrease as the proportion of Sylgard 527 increases relative to Sylgard 184. However, the different forms of these relationships allow for the generation of pairs of formulations that have a similar storage modulus but distinct loss modulus, or *vice versa* (discussed further below). Taken together, these data indicate that we are able to fabricate PDMS formulations with storage moduli ranging from 3 to 130 kPa and loss moduli ranging from 1500 to 3000 Pa, which spans a much broader range of physiological and pathological values than other existing methods. Mean values for each blend are shown in Table 1. To confirm that material properties are not affected by incubation at 37 °C, we repeated rheometric measurements on a representative sample at both room temperature and 37 °C (Fig. S2†). Results indicate no change in mechanical properties. To confirm that material properties are not affected by storage time, we repeated rheological measurements on a representative formulation 6 weeks after the original testing (Fig. S3†). Results again indicate no change in mechanical properties.

**Table 1:** Mean *G*′ and *G*′′ for each formulation. Values are the mean value at a frequency of 100 rad s^−1^

Formulation	*G*′ (Pa)	*G*′′ (Pa)
5 : 1 : 5	126 075	3298
5 : 1 : 10	50 546	3004
5 : 1 : 15	29 498	2642
5 : 1 : 20	15 845	2041
5 : 1 : 30	10 479	2042
10 : 1 : 5	53 014	3786
10 : 1 : 10	13 791	2088
10 : 1 : 15	10 749	2164
10 : 1 : 20	7315	1836
10 : 1 : 30	5210	1764
20 : 1 : 5	11 755	2145
20 : 1 : 10	5773	1748
20 : 1 : 15	4991	1719
20 : 1 : 20	4039	1625
20 : 1 : 30	3814	1686
30 : 1 : 5	6062	1856
30 : 1 : 10	3911	1641
30 : 1 : 15	3553	1654
30 : 1 : 20	3397	1664
30 : 1 : 30	3190	1630

### Protein adsorption onto the surface of PDMS substrates

**Fig. 2:**
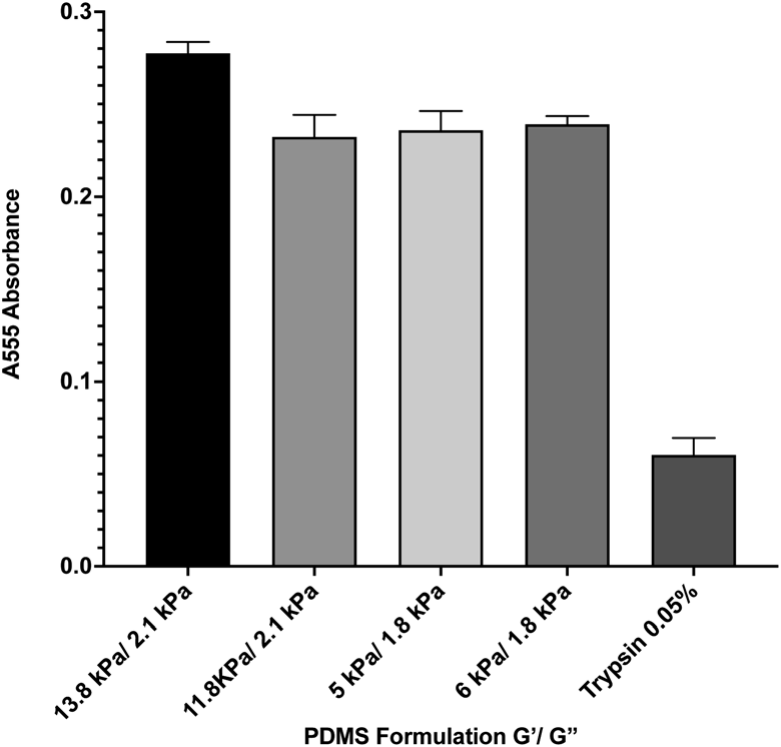
Protein adsorption to PDMS surfaces. Fluorescently-labeled protein was microcontact printed onto 4 representative PDMS formulations, and was then removed *via* trypsin digestion and quantified by spectrophotometry. Results indicate that there is no significant difference between protein adsorption across the formulations. Error bars represent standard error.

During *in vitro* cellular assays, cells cannot typically attach directly to a surface. Instead, they must bind to extracellular matrix proteins that have been adsorbed onto the surface. In order to ensure that protein adsorption was consistent across all formulations, we microcontact-printed substrates with a stamp coated in 200 ng mL^−1^ of the extracellular matrix protein fibronectin that had been labeled with rhodamine isothiocyanate (RITC). Microcontact printing is widely used for facilitating cell attachment onto PDMS substrates, and as such, we utilized this technique as we have previously done.^39^ Coated stamps were brought into conformal contact with UV-treated samples for 30 seconds before peeling. Four substrate formulations that span the range of material properties were selected. Following microcontact printing, substrates were rinsed in PBS, and adsorbed protein was recovered by treating samples with 0.05% of the proteolytic enzyme trypsin, which cleaved attached protein, for 1 hour. Recovered solutions were analyzed for red fluorescence *via* 555 nm absorbance on a spectrophotometer (Fig. 2). A trypsin-only sample was used as a negative control. Results indicate that there was no significant difference in the level of protein attachment to the substrates, regardless of substrate mechanical properties.

### Biocompatibility of PDMS formulations

**Fig. 3:**
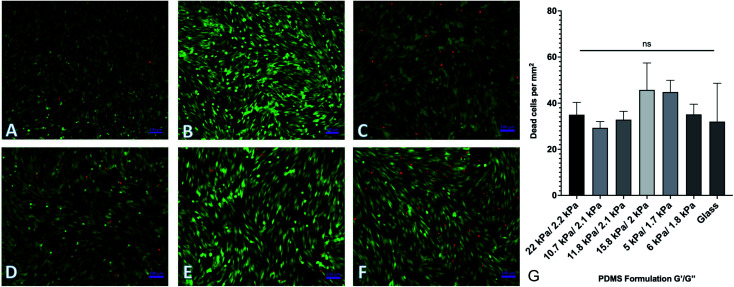
Cell viability on PDMS formulations. Human adipose-derived mesenchymal stem cells were plated onto representative surfaces, cultured for 72 hours, and labeled with a green/red live/dead assay. (A–F) Representative images of labeled cells on (A–F). (G) Quantification of dead cells per mm^2^. Results indicate no significant difference between cell viability on the various substrates. Error bars represent standard error.

By changing the ratios of monomers and crosslinkers in our PDMS formulations, we can fabricate substrates with different material properties. One of the by-products of this is that there are potentially cytotoxic un-crosslinked monomers to which cells could be exposed. In order to test the potential cytotoxicity of the novel formulations, a live-dead assay was performed by seeding 40 000 human adipose-derived mesenchymal stem cells onto each substrate and culturing them for 72 hours. Cells were then rinsed with phosphate buffered saline (PBS) and fluorescently labeled with a solution of 2 μM calcein AM and 4 μM ethidium homodimer III (EthD-III). Calcein AM is plasma membrane permeable and will enter both living and dead cells passively, but only gets activated when cleaved by intracellular esterases. EthD-III labels DNA but is plasma membrane impermeable; as such, only dead cells with compromised plasma membranes can uptake the dye. We imaged labeled cells on an Axiovert inverted fluorescence microscope (Zeiss). Representative images from each of 6 formulations selected from the spectrum of mechanical properties are shown in Fig. 3. Quantified results are shown in Fig. 3G and indicate that the number of living cells vastly outnumbered the number of dead cells, with an average of roughly 40 dead cells per mm^2^. This indicates that any excess monomer or crosslinker in the formulation is insufficient to generate notable cytotoxic effects. Cells were plated onto identically prepared glass coverslips as a control, and results indicate that cell death is similar on glass coverslips to all investigated formulations.

### Development of empirical equations to predict viscoelastic properties

**Fig. 4:**
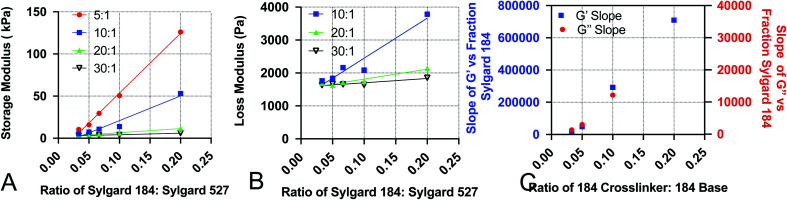
Data used to construct empirical relationships. Empirical relationships were first determined by performing linear regression analysis on (A) the storage modulus *G*′ *versus* the ratio of Sylgard 184 to Sylgard 527 (*p*_184_), and (B) the loss modulus *G*′′ *versus* the ratio of Sylgard 184 to Sylgard 527 (*p*_184_). Finally, the relationship of the slopes determined from A and B to the ratio of 184 crosslinker to 184 base (*p*_cl_) were fit using linear regression. All regression analyses were performed using GraphPad Prism software.

To increase the ease of use for end users of our viscoelastic formulations, we have developed empirical equations that allow users to calculate a predicted value of the loss and storage modulus for a given chemistry. To do this, we performed linear regression analyses on the data acquired for Fig. 1. Analysis was done by performing linear regression on the relationship between *G*′ and the ratio of Sylgard 184 to Sylgard 527 (Fig. 4A) and the relationship between *G*′′ and the ratio of Sylgard 184 to Sylgard 527 (Fig. 4B). A linear regression was then performed on the relationship between the slopes determined from the data in Fig. 4A and B and the ratio of Sylgard 184 crosslinker to Sylgard 184 base (Fig. 4). All regression analyses were performed using GraphPad Prism software. Results indicate that both the loss modulus and storage modulus can be fit to the following equations:

1 *G*′ = *k*_1_(*p*_cl_) × *p*_184_ + *k*^0^_1_(*p*_cl_)

2 *G*′′ = *k*_2_(*p*_cl_) × *p*_184_ + *k*^0^_2_(*p*_cl_)

in which the dependent variable *p*_184_ is the percentage of Sylgard 184 in the formulation. The variables *k*_1_, *k*^0^_1_, *k*_2_, and *k*^0^_2_ are not constant, but are instead functions of *p*_cl_, the percentage of crosslinker in the base : crosslinker ratio of Sylgard 184, and are defined by the following relationships:

3 *k*_1_(*p*_cl_) = *k*_11_ × *p*_cl_ + *k*^0^_11_

4 *k*^0^_1_(*p*_cl_) = *k*_12_ × *p*_cl_ + *k*^0^_12_

5 *k*_2_(*p*_cl_) = *k*_21_ × *p*_cl_ + *k*^0^_21_

6 *k*^0^_2_(*p*_cl_) = *k*_22_ × *p*_cl_ + *k*^0^_22_

**Fig. 5:**
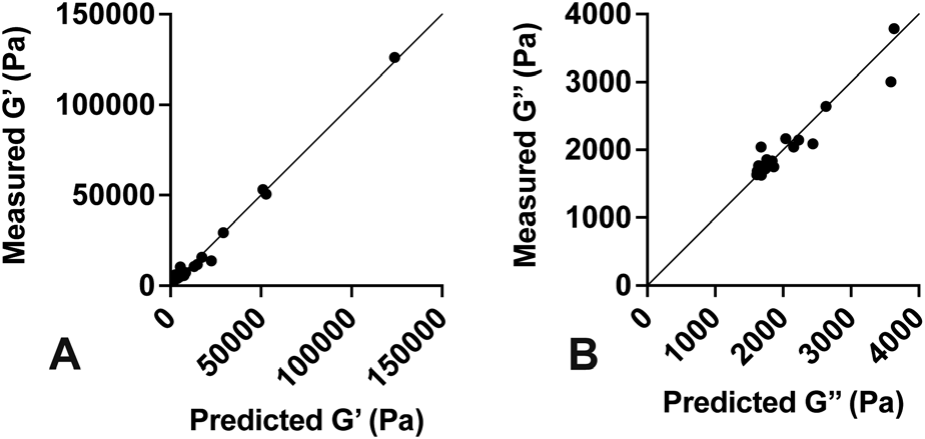
Predicted *versus* measured values of *G*′ and *G*′′. Empirical equations were developed and used to predict (A) *G*′ and (B) *G*′′ for various PDMS formulations. Solid line represents the unity equation, *y* = *x*. Results indicate strong agreement between predicted and measured values.

Values for each constant and *R*^2^ for each equation are shown in Table 2. To quantify the accuracy of the established empirical relationships, we calculated expected values of *G*′ and *G*′′ for each of the formulations measured in Fig. 1. The expected values are plotted *versus* measured values in Fig. 5. Ideally, values should fall along the diagonal, in which the measured and expected values are identical. Results suggest that the mean difference in expected *versus* measured *G*′ was 23.7% ± 4.42%, while the mean difference in expected *versus* measured *G*′′ was 10.1% ± 4.80%.

**Table 2:** *R* ^2^ values and constants for each of the empirical equations used to predict *G*′ and *G*′′ from Sylgard 184 B : C ratio and Sylgard 184 : Sylgard 527 ratio

Equation	*R* ^2^	Constants	
(3)	0.9957	*k*_11_	*k* ^0^_11_
		4.249 × 10^6^	−1.399 × 10^5^
(4)	0.9936	*k*_12_	*k* ^0^_12_
		−1.227 × 10^5^	6372
(5)	0.9991	*k*_21_	*k* ^0^_21_
		1.667 × 10^5^	−4672
(6)	0.9990	*k*_22_	*k* ^0^_22_
		−5159	1754

### Use of empirical equations to predict properties of novel formulations

As a test of the utility of the developed empirical equations, we selected a set of desired *G*′ and *G*′′ values and used the equations to calculate the formulation needed to achieve these properties. These formulations were then generated, and *G*′ and *G*′′ values were measured as described above. Table 3 shows the desired values, the predicted formulation, and the measured *G*′ and *G*′′ values. The empirical equations resulted in a formulation well outside of the parameter space used to generate the equations, and as such, proved a useful experiment in testing their predictive power. Results indicate that measured values were consistent with the desired values, with a mean discrepancy of 22.7% in *G*′ and 41.3% in *G*′′.

## Discussion

Studies in the field of mechanobiology have demonstrated that cells respond to both the storage modulus (stiffness) and loss modulus (viscous component) of an underlying substrate, and that cellular mechanoresponse to these mechanical inputs can dictate cellular proliferation, migration, differentiation, and apoptosis. Studies have also demonstrated that disrupted mechanoresponses can drive disease progression in a host of pathologies. While studies have highlighted the importance of these roles, there are limited experimental tools to generate substrates with varied storage and loss moduli that span physiologically and pathologically relevant values. Here we demonstrate the generation of substrates with varying loss and storage moduli that will allow researchers to assess the independent effects of these two mechanical properties on cellular response. The substrates are shelf stable, biocompatible, and can be fabricated easily by researchers with limited biomaterial fabrication experience. Furthermore, we have developed a set of empirical equations that allows a user to tailor the mechanical properties to their specific needs. As such, this provides a vital new resource in future studies of the effects of storage modulus and loss modulus on cellular responses.

As discussed above, both Sylgard 184 and Sylgard 527 have the same basic chemical structure, but with varying numbers of the repeated units. We hypothesize that it is the mixture of the two bases with differing lengths that generate the unique loss modulus properties of the blends. As the primary resistance to deformation in a polymer is due to chain entanglement, it stands to reason that different concentrations of bases with two distinct lengths will yield different energy loss, and that changing the density of crosslinks will further alter the mechanical properties.

A major benefit of the presented work is that the generated PDMS substrates can be tuned such that a pair of substrates is generated with comparable storage moduli but distinct loss moduli, or *vice versa*. This will allow for future studies in which the effects of storage modulus and loss modulus can be independently isolated. For example, it has been established that human mesenchymal stem cells and hepatic stellate cells both differentiate into osteocytes^40^ or myofibroblasts^41^ when exposed to substrates of increased substrate stiffness. However, a recent study has shown that osteocyte differentiation increased with increased loss modulus,^29^ while another study showed myofibroblast differentiation decreased with increased loss modulus.^27^ It is possible that these cell lines respond differently; however, these studies also used substrates of differing storage modulus, and as such, the independent effects of each variable cannot be isolated. Systems to independently tune the storage and loss modulus, such as the system presented here, will become critical to more deeply explore the effects of viscoelastic properties on cellular responses .

**Table 3:** Predicting *G*′ and *G*′′ for novel formulations using empirical equations

Desired *G*′ (Pa)	Desired *G*′′ (Pa)	Predicted formulation	Measured *G*′ (Pa)	Ratio of measured to desired *G*′	Measured *G*′′ (Pa)	Ratio of measured to desired G′′
35 000	4500	(27.2 : 1) : 0.5	44 938	1.29	7737	1.72
22 000	3000	(25.9 : 1) : 1	28 707	1.31	5058	1.69

## Methods

### Cell culture

ASC52-telo, hTERT immortalized adipose derived mesenchymal stem cells were purchased from American Type Culture Collection and cultured at 37 °C and 5% CO_2_ under standard culture conditions.

### PDMS formulation fabrication

To make the different formulations of PDMS, Sylgard 184 (Dow Chemicals) was thoroughly mixed at a ratio of 5 : 1, 10 : 1, 20 : 1, or 30 : 1 (w/w) base to crosslinker. This mixture was then degassed under vacuum. Separately, Sylgard 527 (Dow Chemicals) was blended at a ratio of 1 : 1 (w : w) base to crosslinker and degassed. After the Sylgard 184 and Sylgard 527 polymers were both mixed thoroughly, Sylgard 184 was mixed with Sylgard 527 at a ratio of 1 : 5, 1 : 10, 1 : 15, 1 : 20, or 1 : 30. Final formulations were cured for 18 hours at 110 °C.

### Spin coating

For cell experiments, prior to curing, 1 mL of the final PDMS blend was pipetted onto 25 mm diameter coverslips that had been cleaned with 70% ethanol. The coverslip was then placed on a Laurell spin processor (Laurell Instruments) using the following parameters: (1) 900 rpm for 10 seconds, (2) 500 rpm for 10 seconds, (3) 300 rpm for 10 seconds, and finally (4) 100 rpm for 15 seconds to generate a coating layer of approximately 100 μm. Coverslips were then cured at 110 °C for 18 hours.

### Rheometry

Parallel plate rheometry was performed using a Discovery HR-3 Hybrid rheometer (TA Instruments) on the PDMS formulations. Samples were prepared as described above and cured in a 65 mL aluminum weighing dish then cut into cylinders 20 mm in diameter with a height of 5 mm. Samples were mounted into the parallel plate rheometer and compressed with an axial force of 3 N. Data was collected at room temperature over a frequency range of strain oscillations from 1 to 100 rad s^−1^ with a 5% strain applied to the samples.

### Protein adsorption

Protein adsorption onto the surface of the different PDMS formulations was tested by first preparing rhodamine-labeled fibronectin by diluting 50 μg mL^−1^ rhodamine-labeled fibronectin (Cytoskeleton Inc) 1 : 20 with 200 ng μL^−1^ fibronectin (R&D Systems). Spin-coated substrates were cleaned in 70% ethanol and then exposed to UV ozone for 10 minutes with a UV ozone cleaner (Novascan) in order to generate reactive oxygen groups to bind protein. Each substrate was then microcontact printed with 100 μL of the labeled fibronectin using a stamp cut to be 1 cm^2^. After microcontact printing, the substrates were placed in 1× PBS for 30 minutes at 37 °C. After 30 minutes, protein was released from the surface with 2 mL of 0.05% trypsin and incubated at 37 °C for another 30 minutes. Absorbance of the collected samples was quantified using a NanoDrop 2000 spectrophotometer (Thermo Fisher).

### Cell viability assay

Substrates for cell viability experiments were prepared as describe above, and rinsed in isopropanol. Substrates were then exposed to UV ozone for 10 minutes as described above, before being incubated with 200 ng μL^−1^ fibronectin for 1 h. 40 000 ASC52-telo human adipose-derived mesenchymal stems cells were seeded onto the substrates. Cells were incubated for 72 hours on the substrates and were then labeled using a cell viability assay (Biotium) per manufacturer's instructions. Briefly, a staining solution of 2 μM calcein AM and 4 μM EthD-III were prepared immediately before imaging. After rinsing twice with 1× PBS, samples were incubated at room temperature in the dark for 30 minutes. After labeling, cells were rinsed again with 1× PBS before imaging.

### Fluorescence microscopy

Fluorescence images of cells from the live/dead assay were imaged on an Axiovert inverted fluorescence microscope (Zeiss). Images were acquired using a 20× objective *via* Zen Blue software (Zeiss). Red cells per image were counted manually and averaged over 25 images.

## Conflicts of interest

There are no conflicts to declare.

## Supplementary Material

RA-011-D1RA03548G-s001
