# Supplementary material for: Rheological characterization of poly-dimethyl siloxane formulations with tunable viscoelastic properties
Source: RSC Adv. 2021 Nov 5;11(57):35910–7. doi: 10.1039/d1ra03548g (PMC9043277; doi:10.1039/d1ra03548g)
Supplement: RA-011-D1RA03548G-s001 [file RA-011-D1RA03548G-s001.pdf]

Electronic Supplementary Material (ESI) for RSC Advances.
This journal is © The Royal Society of Chemistry 2021

Journal Name

ARTICLE TYPE

Cite this: DOI: 00.0000/xxxxxxxxxx

ESI: Rheological Characterization of Poly-dimethyl Siloxane Formulations with Tunable Viscoelastic Properties

Thomas J. Petet, Jr.,[a] Halston E. Deal,[b,e] Hanhsen S. Zhao,[a] Amanda Y. He,[c] Christina Tang,[a] and Christopher A. Lemmon[a,†]

Supplemental Information

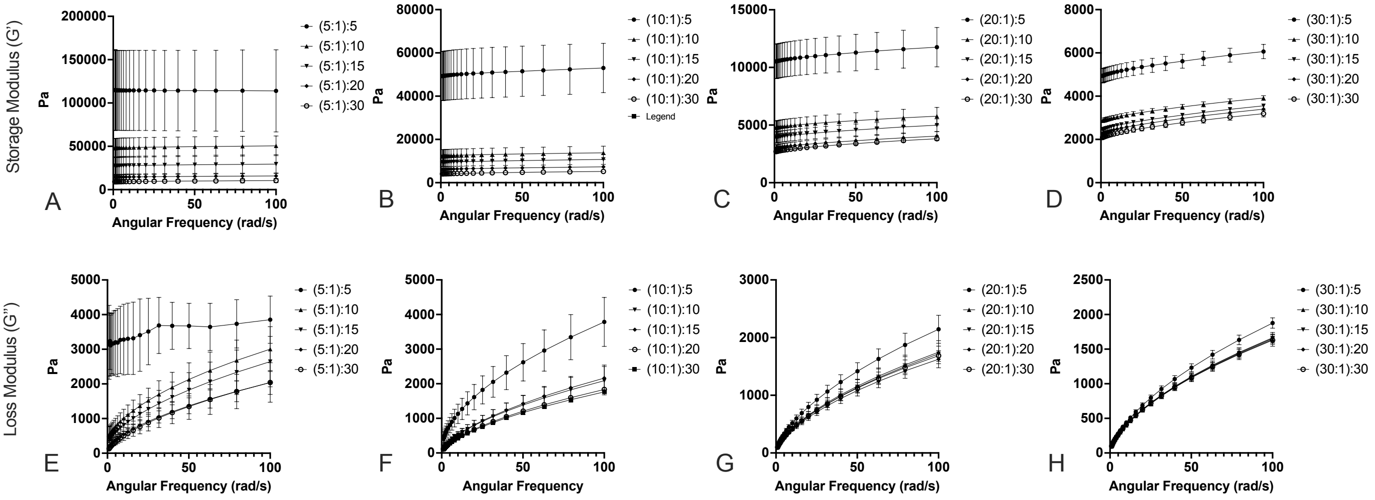

Fig. 1 Mechanical Properties as a Function of Angular Frequency. A-D) Storage modulus (G') versus angular frequency for (A) 5:1 B:C Sylgard 184, (B) 10:1 B:C Sylgard 184, (C) 20:1 B:C Sylgard 184, and (D) 30:1 B:C Sylgard 184. E-H) Loss modulus (G'') versus angular frequency for (E) 5:1 B:C Sylgard 184, (F) 10:1 B:C Sylgard 184, (G) 20:1 B:C Sylgard 184, and (H) 30:1 B:C Sylgard 184. Error bars represent standard error. Formulations are labeld in the legends according to the notation (184B:184C):527.

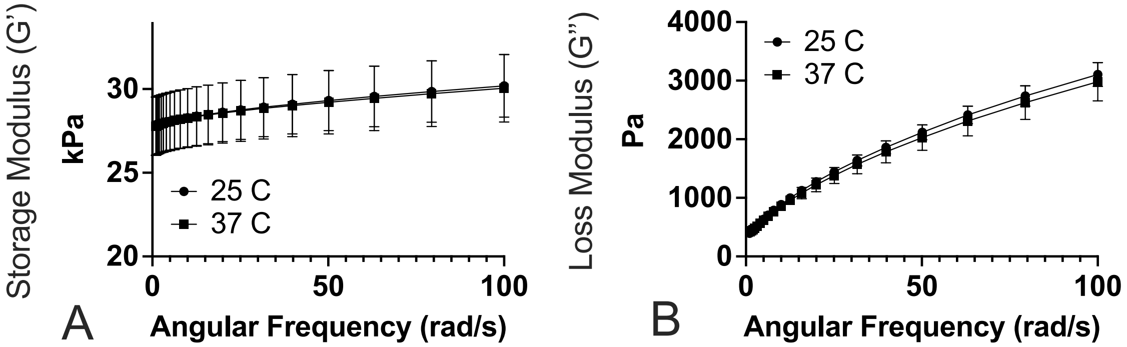

Fig. 2 Effect of Temperature on Mechanical Properties. Mechanical properties of a representative formulation were measured at either 25 °C or 37 °C using a heated stage on the rheometer. Results show that properties do not significantly change at 37 °C. Error bars represent standard error.

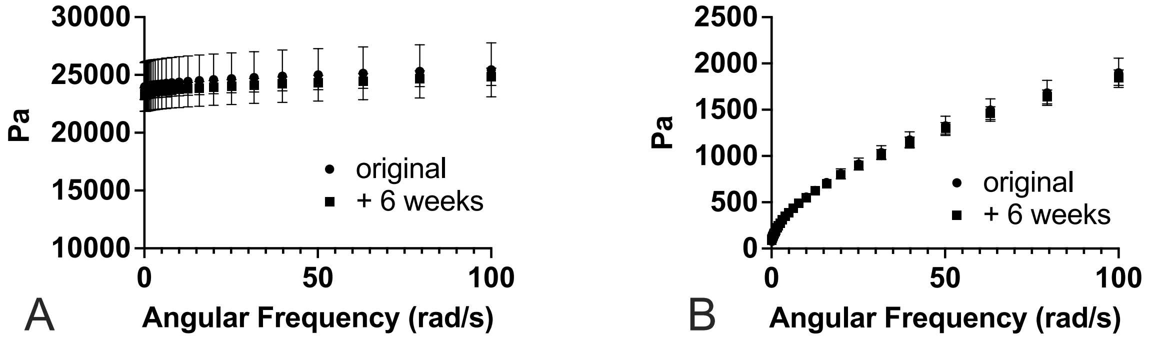

Fig. 3 Changing the Ratios of Sylgard 184 Base:Crosslinker and Sylgard 184:527 creates surfaces with varying storage and loss moduli. A-D) Storage modulus (G') versus decreasing Sylgard 184: Sylgard 527 ratio for (A) 5:1 B:C Sylgard 184, (B) 10:1 B:C Sylgard 184, (C) 20:1 B:C Sylgard 184, and (D) 30:1 B:C Sylgard 184. E-H) Loss modulus (G") versus decreasing Sylgard 184: Sylgard 527 ratio for (E) 5:1 B:C Sylgard 184, (F) 10:1 B:C Sylgard 184, (G) 20:1 B:C Sylgard 184, and (H) 30:1 B:C Sylgard 184. (I) Compiled values for storage modulus for all formulations. (J) Compiled values for loss modulus for all formulations. Error bars represent standard error. Formulations are labeld on the X-axis according to the notation (184B:184C):527.
